# Can adoption of pollution prevention techniques reduce pollution substitution?

**DOI:** 10.1371/journal.pone.0224868

**Published:** 2019-11-07

**Authors:** Sangyoul Lee, Xiang Bi

**Affiliations:** Food and Resource Economics Department, University of Florida, Gainesville, Florida, United States of America; University of Miami, UNITED STATES

## Abstract

Pollution prevention (P2) has become an integral part of the U.S. environmental policy that emphasizes the benefits of preventing pollution generation at the source over treatment or recycling after the generation of wastes. This study extends the existing literature on the effect of voluntary adoption of P2 in reducing toxic wastes by examining the extent to which it reduces pollution substitution. We use facility panel data from the Toxics Release Inventory from 1991 to 2011 to examine the effect of the adoption of P2 techniques on the ratios of water releases to air releases, amounts of treatment to total releases, and amounts of recycling to total releases while controlling for endogeneity of the adoption of P2 techniques and facility fixed effects. We find that the adoption of P2 techniques reduces toxic air and water releases equally, but it is associated with increases in treated and recycled wastes over total releases to the environment.

## 1 Introduction

Pollution controls in the United States rely on command and control regulation that emphasizes end-of-pipe abatement. The problem with this method is that end-of-pipe abatement often focuses on limiting direct releases to a single environmental medium. For example, installing wet scrubbers in smokestacks often turns air pollutants to wastewater, thus solving one environmental problem by creating another [1–3]. Additionally, pollution controls after wastes generation hamper the awareness and ability of firms and workers to identify the root causes of wastes [4]. Recognizing the limitations of end-of-pipe pollution controls, the 1990 Pollution Prevention Act (PPA) sought to shift the emphasis from end-of-pipe controls downstream to pollution prevention upstream by promoting the waste management hierarchy in which pollution prevention (P2) at the source is preferred whenever feasible, followed by recycling, treatment, and releases (including disposal) to the environment.

To provide the public a more comprehensive view of a facility’s waste management hierarchy, the PPA expanded the Toxic Release Inventory (TRI) to include additional reporting on the adoption of P2 techniques and the amount of toxic wastes recycled or reused for energy recovery (incineration) per each TRI chemical [5]. Between 1991 and 2012, a total of 370,000 adoptions of P2 techniques were reported by 21,550 TRI facilities [6].

Existing studies show that, even though many TRI chemicals are not directly regulated by environmental regulations, public access to the TRI publication has motivated polluters to voluntarily reduce toxic releases due to pressures from local communities, investors, regulators, and consumers [7–12]. Recent studies show that voluntary adoption of P2 techniques has contributed to the reduction in toxic releases [6, 13]. However, amid declining toxic releases, total production-related wastes (including amounts of wastes released, recycled, and treated) increased by 40%, from 21.82 billion pounds to 30.57 billion pounds from 2010 to 2017 [5]. Such an increase is partly due to the economic recovery after the 2008 recession [5]. However, given the policy emphasis of using P2 techniques to mitigate the limitation of end-of-pipe pollution controls and to prevent wastes from entering the waste stream for treatment and recycling, there is a need to empirically investigate whether the increase in total toxic wastes can be mitigated by voluntary adoption of P2 techniques.

This examination is particularly important to quantify the overall environmental impact of the voluntary adoption of P2 techniques in the presence of media-specific regulation (such as the Clean Air Act). Media-specific regulation often causes pollution substitution by reducing one type of pollution (e.g., air pollutants) at the expense of another type of pollution (e.g., water pollutants) [1–3,14]. Under pressure to comply with regulation, polluters may increase the use of P2 techniques that are easier to implement with existing end-of-pipe pollution controls (e.g., treatment and recycling), rather than making fundamental changes to prevent toxic materials from entering the waste stream. This casts doubt on the full benefits of P2, for it is bounded by media-specific regulation. Given that few environmental regulations in the United States focus on multi-media emissions, if P2 techniques fail to deliver holistic reductions in toxic wastes, any future U.S. pollution control policy may need to prioritize specific techniques that minimize the “cradle to grave” life cycle of toxic materials.

However, it is difficult to discern substitution by observing declining releases and increasing wastes separately without econometric analysis that controls the change in output. Even if P2 techniques can reduce total production-related wastes, the relative proportions of treated and recycled wastes may still increase while holding total releases constant. In other words, the relative importance of treatment and recycling increases than what it would have otherwise been. This paper empirically examines the effect of voluntary adoption of P2 techniques on pollution substitution by examining the effect of P2 adoption on the ratios of toxic releases versus treatment or recycling of toxic wastes, and of toxic releases to water versus air. In contrast, existing studies on pollution substitution focus on command-and-control regulation, rather than voluntary adoption of P2 techniques. Studies on the adoption of P2 techniques focus on its effect on total toxic releases to the environment [13] or individual type of production-related wastes separately [6] but have not determined pollution substitution among different waste management approaches, such as releases, treatment, and recycling. Additionally, this paper examines the heterogeneous effects by type of P2 techniques in order to inform policy makers on prioritizing P2 techniques that reduce environmental impacts to all environmental media.

We use facility panel data from the Toxics Release Inventory (TRI) for the period 1991 to 2011. The comprehensive nature of TRI allows us to examine two decades of data on P2 technique adoption, toxic releases, and treated and recycled wastes, accounting for about 50% of the U.S. manufacturing base from both public and private facilities [15]. We model the ratio of emissions of one environmental medium to another environmental medium with respect to the cumulative number of P2 techniques adopted, as well as facility specific fixed effects and regulatory and public pressures that could influence a TRI facility’s toxic emissions. Instrumental variables are used to control for endogeneity of the decision to adopt P2 techniques. Our results indicate that the adoption of P2 techniques did not significantly influence substitution between toxic air and water releases, indicating that P2 techniques control all types of releases to the environment equally and mitigate substitution of releases. However, the adoption of P2 techniques on process and equipment modifications is associated with increases in waste treatment and recycling over total releases.

## 2 Background and related literature

The adoption and diffusion of P2 techniques rely on information provisions and voluntary approaches rather than prescriptive technology standards. TRI is an information disclosure tool to help local communities prepare for potential health and environmental hazards caused by the storage, use, and release of toxic chemicals commonly used in manufacturing. Specifically, manufacturing facilities that have 10 or more full-time employees and that manufacture, process, or use any TRI-listed chemicals in amounts exceeding 25,000 pounds per year for manufacturing or processing, or 10,000 pounds per year for otherwise use, must submit annual reports to the Environmental Protection Agency (EPA) on the amounts of toxic wastes released and treated. As part of the voluntary (non-mandatory) policies to encourage P2, TRI was expanded under the PPA to include additional reporting on the adoption of new P2 techniques and the amount of toxic wastes recycled or used for energy recovery (incineration) per each TRI chemical [5].

Earlier studies indicate that there are economic benefits associated with voluntary adoption of P2 techniques. For example, P2 techniques and recycling that allow firms to significantly reduce their waste management costs in compliance with government regulations [16] also significantly contribute to their business profitability [17]. Adopting P2 techniques works well with the total quality environmental management system in improving quality and efficiency [18]. P2 techniques that substitute inputs and modify production processes provide firms a faster payback period than end-of-pipe pollution controls that require fairly large capital investment and management expertise [19, 20]. In addition, studies also indicate that pressures from the public and regulators motivate polluters to voluntarily adopt P2 techniques. Firms often use an environmental management system or P2 programs as a tactical channel to appeal to their stakeholders [21] and to reduce future EPA enforcement actions [22].

Earlier studies find that the adoption of P2 techniques reduced toxic releases, although the magnitude of the effects varied. Harrington, Deltas, and Khanna [13] show that adopting P2 techniques significantly reduced toxic releases by 35–50%, although the effect dissipates within 4~5 years after the first adoption, based on data from TRI facilities that belonged to S&P 500 firms for the period 1991–2001. Bui and Kapon [23] find that state-level P2 programs significantly reduced annual TRI releases by 11–15% through panel analysis on TRI facilities over the period 1988–2003. Bennear [24] shows that facilities subject to management-based regulations achieved greater reductions in total toxic releases and adopted more P2 techniques (the author infers that the reductions may have been mainly achieved through P2 techniques). Ranson et al. [6], examining the effects of P2 techniques on total releases, treated wastes, and recycled wastes separately, find that between 1991 and 2012, a total of 370,000 adoptions of P2 techniques were reported by 21,550 TRI facilities and contributed to the reduction of direct toxic releases by 9–16% for all TRI facilities.

Previous empirical studies examining pollution substitution focused on regulation, rather than on voluntary P2 techniques. Gibson [2] shows that facilities regulated by the Clean Air Act (CAA) transfer pollutants from air to water and to unregulated facilities within the same parent company. Bi [1] shows that stricter air regulations induce coal-fired power plants to shift air pollutants to waterways and landfills. In contrast, Sigman [25] finds little evidence of pollution substitution between wastes disposal and air emissions that use chlorinated solvents, although higher costs of waste disposal caused air emissions to increase. Greenstone [3] models CAA-regulated toxic releases to air, water, and land in the iron and steel industry as a function of a county’s nonattainment designation and shows that a county’s nonattainment designation decreased emissions across all media.

This study builds on the existing studies on pollution substitution and voluntary adoption of P2 techniques but differs from the previous literature in three ways. First, the previous literature focuses on the effect of pollution substitution induced by environmental regulations, particularly the CAA (e.g., [1–3]), rather than voluntary adoption of P2 techniques. Second, the existing studies focus on the effect of P2 techniques in reducing total toxic releases [6,13] rather than on the ratios of pollutants across media pathways, and therefore are unable to determine pollution substitution. Additionally, previous research finds that P2 techniques, ranging from making improvements in equipment and raw materials to inventory controls, have differential impacts on total toxic releases [6]. However, none has examined the effects of various types of P2 techniques on pollution substitution. This study separates P2 techniques into three categories and examines their effects on pollution substitution to identify P2 techniques that minimize wastes.

## 3 Model

We model multiple pollutants (e.g., releases to air and to water) as inputs used by a TRI facility for producing output, following the previous literature [2, 26, 27]. Modeling pollutants as inputs follows the rationale that releasing pollutants entails explicit costs of violating regulations or implicit costs of adverse publicity and that a profit maximizing facility will choose the optimal ratio of polluting inputs to minimize these costs. For example, parts of the costs include compliance to the existing regulations, due to the overlap between some of the TRI chemicals and the existing regulations. Our study focusses on 296 TRI chemicals and 151 of them are also classified as hazardous air pollutants or contributing to criteria air pollutants subject to emission standards or national ambient air quality standards [28].

Following Gibson [2], we assume a TRI facility has two polluting inputs, *R* and *W*, and another input, *L* (such as labor). The cost function can be expressed as *C* = *f*(*Q*)*C(*P*_*R*_,*P*_*w*_,*P*_*L*_), in which *f*(*Q*) represents the output quantity, and *P*_*R*_, *P*_*w*_, and *P*_*L*_ represent the cost of pollution inputs *R* and *W*, and the cost of *L*, respectively. The facility operates in competitive input and output markets, and the cost function is homogenous degrees of one in all prices. In other words, if all prices double, the total costs will double, and the optimal allocation of inputs derived from cost minimization does not change.

As a result of cost-minimization, the conditional input demand for one type of pollutant can be obtained by differentiating the above equation with respect to its price, such that $W^{*}=f(Q)*\frac{\partial C}{\partial P_{W}};R^{*}=f(Q)*\frac{\partial C}{\partial P_{R}}$. The conditional input demand functions are homogenous degrees of zero in prices. The ratio of the two optimal inputs demands can be represented with ratios of the input prices:

$$\frac{W^{*}}{R^{*}}=\frac{\frac{\partial C}{\partial P_{W}}}{\frac{\partial C}{\partial P_{R}}}=h\left(\frac{P_{R}}{P_{W}},\frac{P_{L}}{P_{W}},\frac{P_{L}}{P_{R}}\right)$$ (1)

Estimating the ratio of optimal inputs with respect to the price proxies reveals the net substitution between the inputs and the estimation is independent of output quantity and does not need to include the prices of all inputs, such as *P*_*L*_ [2]. Net substitution with respect to price change in pollutant *R* is expressed as the percentage change in pollutant *W* with respect to price change in pollutant *R* minus the percentage change in pollutant *R* in response to price change in pollutant *R*, holding output level fixed [2]. It is more relevant than gross substitution in policy analysis on pollution substitution because gross substitution also includes the input demand response to change in output.

Following this theoretical insight, our empirical model proceeds by modeling the ratio of pollutants as described in Eq (2) below:

$${ln(ratio)}_{it}=a_{i}+X_{it-1}^{\prime }\beta_{1}+\beta_{2}{pp}_{it-1}+D^{\prime }\delta_{1}+(D\prime \times t)\delta_{2}+u_{it}$$ (2)

The dependent variable, ln(*ratio*)_*it*_, is defined as facility *i’*s log ratio of two pollutants at year *t*. *a*_*i*_ represents unobserved facility specific fixed effects. *pp*_*it*−1_ is the variable of our interests, and measures the intensity effect of P2 techniques on the implicit prices of the two pollutants by affecting costs to comply with regulations or to appeal to the public.

Gibson [2] shows that the coefficient *β*_2_ can be expressed as a scalar function, *β*_2_ = *νσ*, where σ is the elasticity of net substitution of the two pollutants with a scalar *ν*. A negative sign of *σ* indicates the two pollutants are net complements and a positive sign indicates the two pollutants are net substitutes (i.e., holding output fixed). *ν* is the percentage increase in relative prices of two inputs ($\frac{P_{R}}{P_{W}}$) induced by one incremental adoption of P2 techniques. If P2 activities successfully caused holistic reduction in all emissions, adopting P2 techniques will not incur pollution substitution among the environmental media, thus estimates of *β*_2_ are expected to be zero.

Additionally, *X*′_*it*−1_ represents a vector of facility *i*’s covariates affecting pollution substitution in the preceding year. *D*′ represents a vector of fixed effects that include state and industry (defined by the 2-digit SIC codes) dummies. These dummy variables control for unobserved factors common for a given state or a given industry that affect the pollution ratios. *D*′×*t* represents the interactions between state and industry dummies and the linear year trend to control for state and industry specific year trends, such as industry’s technology change and state’s environmentally friendly efforts, which could affect pollution substitution.

Although facility specific fixed effects can be controlled in estimating (2) by using fixed-effects panel models, *cumulative P2* may still be endogenous. For example, a facility or its parent firm may have adopted environmental management systems that have been shown to increase the adoption of P2 techniques [4,18]. Such systems may also improve chemical-use efficiency and reduce pollution to all environmental media. Since these factors are unobservable to us and are likely to be time-varying within the two decades of the sample period, fixed-effects estimates on Eq (2) are likely to biased.

Thus we use 2SLS panel regression with instrumental variables and multiple levels of fixed effects that takes into account the correlation of the error terms across time to estimate Eq (2) [29]. The first-stage regression is shown in Eq (3) below, in which *Z*′_*it*−2_ represents the vector of two time-varying instrumental variables that are lagged by two years.

$${pp}_{it-1}=\gamma_{0}+X_{it-1}^{\prime }\gamma_{1}+{Z\prime }_{it-2}\gamma_{2}+D^{\prime }\gamma_{3}+(D\prime \times t{)\gamma }_{4}+v_{it-1}$$ (3)

These two instrumental variables are excluded in the second stage of estimating Eq (2). They are expected to be correlated with time-varying *pp*_*it*−1_ but are uncorrelated with time-varying unobservables, such as adoption of the environmental management system in Eq (2), except through their effects on *pp*_*it*−1_, conditional on other covariates. We discuss the choice of the instrumental variables in details in section 3.2.

### 3.1 Variable construction

We estimate (2) for each one of the ratios: toxic releases to water versus releases to air, the amount of toxic recycling wastes versus total toxic releases, and the amount of treated toxic wastes versus total toxic releases, respectively. This analysis focuses on a group of TRI toxic chemicals (296) listed from 1988 onwards without any change in reporting requirements [28]. We refer to the 296 toxic chemicals as the “*core chemicals*” hereafter. These three log ratios are generated using the inverse hyperbolic sine function that is similar to logarithm transformation and allows retaining zero-valued observations by transforming a variable (*x*) into ($ln(x+\sqrt{x^{2}+1})$ [30]. To generate the three ratios, we aggregate the annual amount of total toxic releases, recycled wastes, treated wastes, P2 techniques, water, and air releases for *core chemicals* for each TRI facility. Specifically, the amount of total releases to water, air, and land onsite, and the amount of toxics transferred to disposal offsite. We did not examine the ratio for land to air releases separately, as 95% of the sample did not report direct onsite releases to land.

We construct the variable *pp*_*it*−1_ to approximate the intensity of P2 techniques implemented since a facility first became an adopter of P2 techniques because TRI reporting requirement applies only for incremental (new) P2 techniques per TRI chemical in a given year. Specifically, we first aggregate the cumulative number of P2 techniques reported by a facility for the *core chemicals* up to one period prior, and then divide the cumulative number by the total number of reported *core chemicals* by each facility up to one period prior. We refer to this new variable as “*cumulative P2*” hereafter. Considering TRI provides annual data, we lag the *cumulative P2* variable to avoid contemporaneous problems with the dependent variable. Nevertheless, a TRI facility may discontinue any previously reported P2 techniques without having to report disadoption. We conduct robustness checks on the definition of the *cumulative P2* variable in the next section.

Following the previous literature, we use environmental inspections by state and federal regulators on the facilities and each county’s attainment status as proxy variables for the regulatory pressure for a TRI facility. Hanna and Oliva [31] show that an actual inspection under the CAA reduces a firm’s air emissions by 15% for the next five years using the event study method. If CAA inspections increased the compliance cost to CAA, we would expect facilities to reduce air releases more than other types of emissions, such as water releases and wastes for treatment and recycling. We aggregate the annual number of inspections by federal and state agencies at the facility level from EPA’s Aerometric Information Retrieval System (AIRS) Facility Subsystem (AFS) database and use the unique facility level identifier to link the AFS dataset with the TRI. We do not include inspections under the Clean Water Act (CWA) in our empirical model because CWA focuses on conventional water pollutants instead of toxic water pollutants.

We use a county’s nonattainment status as another variable for regulatory pressure. Under the CAA, the National Ambient Air Quality Standards (NAAQS) for carbon monoxide, sulfur dioxide, total suspended particle pollution (PM), ground-level ozone, nitrogen oxide, and lead are set by the EPA, which is responsible for ensuring that ambient air quality in each county meets the NAAQS [32]. Once a county exceeds any of the standards for a pollutant, the county is designated a nonattainment area for the particular pollutant. In order for a nonattainment county to meet the standard, the state government needs to develop a state implementation plan. Once EPA approves the plan, facilities in the nonattainment county have to adopt pollution control methods to reach attainment status. Following the most recent literature, we expect that facilities in nonattainment counties would reduce more air releases than other types of toxic emissions.

We focus on attainment status for two types of pollutants, particulate matter and ground-level ozone (most counties obtained attainment status for the rest of the criteria for air pollutants by 1998). We define *PM Nonattainment* and *Ozone Nonattainment* as dummy variables that are equal to 1 if a county falls into nonattainment with PM or ozone, respectively, in a given year, and 0 otherwise. Following previous studies, the change in a county’s attainment status is considered exogenous to a facility’s toxic releases and the two variables are lagged by one period in the estimations to allow time for counties and TRI facilities to respond to the regulation [1–3]. Based on the location information of the TRI facilities, we merge the TRI data with the county’s annual nonattainment status in compliance with the NAAQS from EPA’s Green Book [33].

Natural log of *the number of employees* of the facility is included in the covariate vector to control for the size of the facility. TRI does not include information regarding the level of production and the number of employees, so we use the number of employees obtained from the National Establishment Time-Series [34] to control for the size of the facility. To take into account the pressures from local communities to reduce toxic pollution, we use the annual unemployment rate of the county in which a facility is located from the U.S. Bureau of Labor Statistics [35].

### 3.2 Instrumental variables

We apply two time-varying instrumental variables (IVs) based on findings from the previous literature. They are the lagged proportion of facilities that have adopted P2 techniques in the same industry (defined by the 2-digit SIC codes), excluding the facility itself, and the lagged average number of federal inspections on other facilities, excluding the facility itself, in the same state. Both IVs are lagged by two periods.

Both IVs are expected to be strongly correlated with the number of P2 techniques. Specifically, a facility is more likely to adopt P2 if others in its industry or its state peers have adopted P2 techniques, either through information spillovers or through shared supply chains. For example, Harrington [36] finds that a facility’s decision to adopt P2 techniques can be affected by the trend of adopting P2 by other facilities in the same industry. Sam [22] finds positive correlation between the number of P2 techniques adopted and the number of state-level environmental inspections. Chang and Sam [37] examine whether greater numbers of P2 techniques adopted led to greater numbers of environmental patents. They correct the endogeneity problem by using the frequency of inspection by both federal and state authorities on each facility three years prior as the IVs. We expect that the increasing regulatory pressure on peer facilities in the same state is likely to increase the adoption of P2 techniques by peer facilities. This is likely to increase a facility’s own adoption of P2 techniques through learning from peer facilities within a state.

The valid IVs have to satisfy the exclusion restriction. That is, conditional on the covariates, the IVs should have no effect on pollution substitution in the current period except through a facility’s own adoption of P2 up to the previous period. We believe these IVs satisfy the exclusion restriction because they are based on the production circumstances of other facilities two periods prior. Additionally, the number of inspections on other facilities for compliance with the Clean Air regulation two periods prior depends on their violation history, state budget of enforcements, and local complaints. While more inspections may motivate facilities from the same state to adopt P2 techniques through deterrence, they are unlikely to influence a facility’s pollution substitution after controlling for environmental inspections and other regulatory pressures on the facility itself. Summary statistics of the explanatory and instrumental variables are reported in Table 1.

**Table 1:** Summary statistics.

Variable		Mean	Std. Dev.	Min	Max
**Dependent Variables**					
	***<logarithm (inverse hyperbolic form)>***				
	Treatment/Total release	0.85	2.03	0.00	15.79
	Water/Air	0.09	0.60	0.00	11.13
	Recycling/Total release	1.70	3.01	0.00	16.10
	Offsite recycling/Total release	1.45	2.91	0.00	15.96
**Explanatory Variables**					
	Cumulative P2 (normalized by number of chemicals)	2.52	6.15	0.00	251.00
	Total number of inspection (both federal & state)	0.28	0.63	0.00	96.00
	PM nonattainment (1 = nonattainment for PM, 0 = otherwise)	0.20	0.40	0.00	1.00
	Ozone nonattainment (1 = nonattainment for Ozone, 0 = otherwise)	0.41	0.49	0.00	1.00
	Unemployment rate (%) in county	5.91	2.36	0.99	31.11
	Number of employees (log)	4.79	1.30	0.69	10.20
	Production ratio	1.02	0.298	0.005	2.995
**Instrument Variables**					
	Share of facilities adopting P2 in the same industry	0.53	0.17	0.00	1.00
	The average number of inspections on other facilities in the same state	0.28	0.22	0.00	1.80

Note: Number of Observations = 190,395; Number of Unique Facilities = 21,650; Year = 1991 to 2011.

## 4 Results

### 4.1 Main results

We report the estimated effects of cumulative P2 adoption on different types of pollution substitutions (Table 2), including total amount of wastes treated to total releases, total amount of water releases to total air releases, and total amount of wastes recycled to total releases. We also report the first-stage regression results in column 1 and the test statistics on the validity of instrumental variables (rows 10–11). The higher share of facilities that had adopted P2 techniques in the same industry two periods prior is associated with the higher level of a facility’s lagged cumulative P2 (column 1, row 1). Though the lagged average number of inspections on other facilities in the same state alone did not significant influence a facility’s lagged cumulative P2, both instruments are jointly strongly correlated with the endogenous variable as indicated by the statistically significant F-statistics (F = 26.768) [29,38]. None of the Hansen’s J statistics for orthogonality of the instruments is statistically significant at the 5% significance level, indicating the instruments are orthogonal to the error terms (rows 10–11).

**Table 2:** Two–Stage fixed–effects estimation of effects of P2 on pollution substitutions.

Variable	First Stage	Second Stage		
	(1)	(2)	(3)	(4)
	Cumulative P2(t-1)	Treatment/ Releases	Water/Air	Recycling/ Releases
*Excluded IVs*				
Proportion of facility adoptions of P2 in same industry (t-2)	3.519***			
	(0.494)	—	—	—
Avg. number of inspections on other facilities in same state (t-2)	-0.140			
	(0.129)	—	—	—
*Covariates*				
Cumulative P2 (t-1)	—	0.086**	0.013	0.170***
		(0.041)	(0.017)	(0.057)
Total inspections (t-1)	0.031	0.008	-0.001	-0.002
	(0.019)	(0.012)	(0.003)	(0.008)
PM nonattainment (t-1)	-0.031	0.043	0.010	0.040
	(0.140)	(0.031)	(0.012)	(0.054)
Ozone nonattainment (t-1)	-0.093	0.038*	-0.003	0.105***
	(0.083)	(0.021)	(0.008)	(0.035)
Unemployment rate (%) (t-1)	-0.005	0.002	-0.002	0.004
	(0.012)	(0.003)	(0.001)	(0.005)
No. of employees (log) (t-1)	0.110**	-0.014	-0.009*	-0.001
	(0.048)	(0.012)	(0.005)	(0.019)
Over-identification (Hansen’s J)	—	0.187	0.037	1.182
P-value of Hansen’s J	—	0.665	0.847	0.277
R-squared	0.706	0.768	0.595	0.747

Notes: N = 132,445, Number of facilities = 13,754.

*** p<0.01

** p<0.05

* p<0.1.

Robust standard errors in parentheses (clustered by facility). All models control for facility specific fixed effects in addition to state and industry fixed effects and their interactions with linear year trends. For all second–stage models, Hansen’s J statistics indicate the orthogonality of the instrumental variables cannot be rejected. The analysis is from 1993 as a result of using two years’ lagged IVs. Weak instrument test statistics is 26.768, representing a statistically strong correlation between endogenous variables and IVs, given the Wald F statistic based on the Kleibergen–Paap rk statistics in the presence of clustered standard errors.

We find that the ratio of treated wastes versus total releases increased as facilities adopted more P2 techniques (Table 2, column 2, row 3). The coefficient on the *cumulative P2* techniques was 0.083 and was statistically significant at the 5% significance level. Based on this information, one additional P2 technique adopted per chemical increased the amount of treatment in the current period by 8.3%, holding total releases and other covariates fixed. To put this into context, the average total releases were 58,235 pounds, the average quantities of treated wastes were 59,582 pounds, and the input ratio of treated wastes to total releases was 1.02 in 1991. Holding the covariates and total releases at their averages in 1991, adopting one additional P2 technique per chemical increased the ratio of the two pollutants to 1.10. Such an increase was equivalent to an increase of 4,945 pounds in the amounts of treated wastes. The positive correlation between end-of-pipe treatment and adoption of P2 techniques is also noted in Dutt and King [39]. They suggest that measuring wastes more precisely through treatment allows workers and managers to better identify P2 opportunities. Similarly, our results suggest that facilities use P2 techniques in combination with treatment to reduce total releases.

Similarly, we find that facilities that adopted greater *cumulative P2* techniques had greater substitution between recycling and total releases (Table 2, column 4). The adoption of one additional P2 technique per chemical significantly increased the ratio of recycled toxics to total releases by 17%, holding other variables constant. To put this coefficient into context in 1991, the amounts of recycled toxics was 102,803 pounds, and its ratio to total releases was 1.77. Based on the estimated coefficient on *cumulative P2* in column 3 of Table 2, an additional adoption of P2 techniques per chemical increased the ratio by 17%. This ratio changed to 2.07 following an adoption of a P2 technique, holding covariates and the toxic releases at their average levels in 1991, equivalent to an increase of 17,477 pounds of recycled toxic wastes. The larger impact of adopting P2 techniques on recycled compared to treated wastes can be the result of the joint use of recycling methods and P2 techniques. For example, Cagno, Trucco, and Tardini [17] note that the adoption of P2 techniques is often accompanied by onsite recycling that modifies the flow of the waste stream and recovers wastes to be used by other processes within a facility. This saves a facility’s costs to process additional raw materials and to dispose of hazardous wastes.

We find no statistical evidence that adopting more P2 techniques is associated with greater pollution substitution between water releases and air releases (Table 2, column 3). This implies that adopting P2 techniques affects both types of direct releases to the environment equally, which suggests that using voluntary P2 techniques can overcome the problem of direct releases substitution across environmental media often induced by regulation.

The estimated impacts of other covariates are consistent with our expectations and previous studies. The nonattainment designation of ground-level ozone in a county is associated with an increase of 3.8% and 10.5% in the ratios of treatment to total releases and recycling to total releases, respectively (Table 2, row 6). This positive effect of ground-level ozone nonattainment status on pollution substitution is consistent with Bi [1]. However, a county’s attainment status with respect to particulate matter does not significantly influence substitution between treatment and total releases (Table 2, row 5). The different results between the two types of nonattainment status may be due to the intrinsically different abatement approaches adopted by facilities to abate particulate matter and ground-level ozone pollutants. For example, Bi [1] finds that coal-fired power plants increased wastes transferred for recycling and treatment in order to comply with the CAA for ground-level ozone pollutants by reuse and recover solutions for pollution abatements. Compliance with PM emission standards can occur through reducing particulate matter from air stacks by capturing flying ashes, which increases releases to landfills or ash ponds. Furthermore, we do not find that regulatory pressures in terms of the number of EPA inspections affect pollution substitution significantly (Table 2, row 4). This is consistent with Bi [1] in which CAA inspections did not significant affect the amounts of toxic wastes by coal-fired power plants.

### 4.2 Results by types of P2 techniques

The effects of P2 techniques on a facility’s pollution substitution may vary by types of techniques. Currently, TRI facilities can report up to 43 types of P2 techniques, categorized into eight types: (i) good operation practices (e.g., improving recordkeeping and production scheduling), (ii) inventory control, (iii) spill and leak prevention, (iv) raw material modification, (v) process modification, (vi) cleansing and decreasing modification, (vii) surface and finishing modification, and (viii) product modification. Among the eight types of P2 techniques, Ranson et al. [3] find that raw material modification had the largest negative impact on toxic releases, whereas changes in inventory control and operating practice did not significantly reduce toxic releases. Sam [22] further categorizes the eight types into three groups: operational procedure modification (e.g., good operation practice and inventory control), material modification (e.g., changing raw materials and product designs), and equipment modification (e.g., spill and leak prevention and improving cleanup process). He finds that the effects of adopting P2 techniques on subsequent environmental violations differ by types of P2 techniques. Only operational procedure modification (i.e., P2 types i and ii) reduced violations for all facilities.

To investigate the heterogeneous effects by types of P2 techniques on pollution substitutions between treatment and releases and between recycling and releases, we separate the *cumulative P2* variable into three types of P2, following Sam [22], to examine their effects on the three ratios, respectively (Table 3). Variable *P2 PROC* represents techniques related to changes in operating procedure (P2 types i and ii); variable *P2 EQUIP* represents techniques on the installation of environmentally friendlier equipment and processes (P2 types iii, v, vi, and vii); and variable *P2 MAT* represents techniques involved in raw material and product modification (P2 types iv and viii).

**Table 3:** Second–Stage fixed–effects panel estimates on pollution substitution by types of P2 techniques.

Variable	Dependent Variables		
	(1)	(2)	(3)
	Treatment / Releases	Water / Air	Recycling / Releases
Cumulative P2 PROC (t-1)	0.028	0.003	0.057
	(0.047)	(0.014)	(0.061)
Cumulative P2 EQUIP (t-1)	0.100*	0.017	0.216***
	(0.051)	(0.021)	(0.071)
Cumulative P2 MAT (t-1)	0.105	0.010	0.172*
	(0.072)	(0.036)	(0.104)
Total number of inspections (t-1)	0.008	-0.001	-0.002
	(0.012)	(0.003)	(0.008)
PM nonattainment (t-1)	0.046	0.010	0.043
	(0.031)	(0.012)	(0.053)
Ozone nonattainment (t-1)	0.036*	-0.003	0.101***
	(0.020)	(0.008)	(0.034)
Unemployment rate (%) (t-1)	0.002	-0.001	0.004
	(0.003)	(0.001)	(0.005)
Number of employees (log) (t-1)	-0.013	-0.008*	0.001
	(0.012)	(0.005)	(0.018)
Weak instrument (Wald F-stat.)	15.671		
Over-identification (Hansen’s J)	0.129	0.054	1.545
P-value of Hansen’s J	0.676	0.825	0.209
Adjusted R-squared	0.796	0.639	0.781

Notes: N = 132,445, Number of facilities = 13,754.

*** p<0.01

** p<0.05

* p<0.1.

Robust standard errors in parentheses clustered by facility. All models control for facility specific fixed effects in addition to state and industry dummies and their interactions with linear year trend. Hansen’s J statistics indicate the orthogonality of the instrumental variables (proportions of facilities adopting the three types of P2s in the same industry and average number of inspections on other facilities in the state two years prior) cannot be rejected. The analysis is from 1993 as a result of using two years lagged IVs. Weak instrument test presents a statistically strong enough correlation between endogenous variables and IVs, given the Wald F statistic based on the Kleibergen–Paap rk statistics in the presence of clustered standard errors. But statistical significance for the first–stage F statistics is not determined because the critical value of weak IV test for three endogenous variables corrected for size distortion is not available [37].

Because all three types of P2 techniques are potentially endogenous, we have recreated three additional instruments based on the percentage of other facilities in the same industry (i.e., defined by the 2-digit SIC codes) that adopted the three specific types of P2 techniques (i.e., procedure modification, material modification, and equipment modification) two years prior. For each model reported in Table 3, the instrumental variables are percentages of facilities that adopted P2 *PROC*, *P2 EUIP*, and *P2MAT* in the same industry two years prior, and the average number of federal inspections on other facilities in the same state two years prior.

We find that P2 techniques related to equipment and process modification (*P2 EQIP*) significantly increased the ratio of treated versus total releases by 10% (statistically significant at 10%) and recycled versus total releases by 21.6% (Table 3, columns 1 and 3, row 2). P2 techniques related to procedure modification *(P2 PROC)* did not significantly affect any type of pollution ratios. P2 techniques related to material modification *(P2 MAT)* only increased the ratio of recycling versus total releases (statistically significant at 10%).

The results in Table 3 suggest that procedure modifications reduced all types of toxic wastes equally, while P2 techniques involving modifying equipment (e.g., modifications in spill prevention, cleaning, degreasing, and surface preparation) increased wastes to be treated or recycled downstream relative to total releases. Ranson et al. [6] find that P2 techniques involving raw material and product modifications had the largest marginal impact on reducing toxic releases. They argue that changing raw materials and products are more complex and resource intensive, thus more effective in reducing actual pollution. Additionally, previous case studies show that firms often fail to identify waste materials for treatment or reuse in the downstream process unless they make changes in materials, procedures, and equipment [40,41]. Our results suggest that changes in raw materials, product designs, and procedures should have priority over changes in equipment and processes to mitigate pollution substitution induced by P2 techniques.

### 4.3 Robustness check

We conduct several robustness checks. First, we include the variable *production ratio* reported in the TRI to control for changes in production scale at the facility level. This ratio of the current year’s output versus last year’s output contributed by a TRI chemical is reported by TRI facilities for each TRI chemical. This ratio is chemical-specific and should be reported as a decimal ratio if reported correctly. Previous studies indicated some facilities reported negative numbers or omitted decimals in reporting [39]. Following previous studies, we removed the top and bottom 1% as outliers and took the median value of the reported production ratios for all core chemicals for each TRI facility in a given year [42] and included it as an additional covariate. The results are reported in Table 4. We find that the production ratio is positively correlated with the amounts of treatment and recycling versus total releases. Nevertheless, the main conclusions are robust to adding this additional variable.

**Table 4:** Second–Stage fixed–effects panel estimates on effects of P2 on pollution substitutions with production ratio.

	Dependent Variables		
	(1)	(2)	(3)
	Treatment / Releases	Water / Air	Recycling / Releases
Cumulative P2 (t-1)	0.066*	0.008	0.149***
	(0.037)	(0.015)	(0.050)
Total number of inspections (t-1)	0.008	-0.002	-0.004
	(0.011)	(0.004)	(0.008)
PM nonattainment (t-1)	0.058*	0.008	0.040
	(0.031)	(0.012)	(0.053)
Ozone nonattainment (t-1)	0.033	-0.003	0.113***
	(0.021)	(0.008)	(0.035)
Unemployment rate (%) (t-1)	0.000	-0.001	0.005
	(0.003)	(0.001)	(0.004)
The number of employee (log) (t-1)	-0.009	-0.007	0.002
	(0.012)	(0.005)	(0.018)
Production ratio (t-1)	0.048***	0.006	0.051**
	(0.014)	(0.005)	(0.021)
Weak instrument (Wald F-stat.)	30.708***		
Over-identification (Hansen’s J)	0.452	0.143	2.036
P-value of Hasen’s J	0.501	0.705	0.154
Adjusted R-squared	0.783	0.604	0.764

Notes: N = 122,033, Number of facilities = 13,186.

*** p<0.01

** p<0.05

* p<0.1.

The number of observations was reduced to 122,033 since none all facilities reported valid production ratios. Robust standard errors in parentheses (clustered by facility). All models control for facility–specific fixed effects, in addition to state and industry fixed effects and their interactions with linear year trends. For all models, the Hansen’s J statistics indicate the orthogonality of the instrumental variables (proportion of facilities adopting P2 in same industry and average number of inspections on other facilities in the state two years prior) cannot be rejected. Weak instrument test presents a statistically strong enough correlation between endogenous variable and IVs, given the Wald F statistic that is based on the Kleibergen–Paap rk statistics in the presence of clustered standard errors.

Second, TRI reports only incremental (new) P2 techniques on an annual basis. A previous study suggests that the effectiveness of P2 techniques in reducing toxic releases diminishes after 5 years [12]. Therefore, to avoid over-estimating the effect of cumulative P2, in contrast to accounting for P2 techniques since 1991 up to the preceding year, we only include the total number of P2 techniques adopted in the most recent six years up to the preceding year as an alternative definition for the *cumulative P2* variable. The results are reported in Table 5. The main conclusions are consistent with this alternative definition o*f cumulative P2* variable, though the magnitude of the estimates on *cumulative P2* is reduced. The effect on ratio of treatment to releases is reduced from 8% to 4% and the effect on the ratio of recycling to releases is reduced from 16% to 8%.

**Table 5:** Second–Stage fixed–effects panel estimates on effects of P2 on pollution substitutions using cumulative P2 techniques from recent five years.

Variable	Dependent Variables		
	(1)	(2)	(3)
	Treatment / Releases	Water / Air	Recycling / Releases
Recent 5 years’ Cumulative P2 (t-1)	0.037**	0.006	0.079***
	(0.018)	(0.008)	(0.023)
Total number of inspections (t-1)	0.011	-0.000	0.003
	(0.012)	(0.003)	(0.007)
PM nonattainment (t-1)	0.039	0.010	0.032
	(0.029)	(0.012)	(0.048)
Ozone nonattainment (t-1)	0.034*	-0.003	0.097***
	(0.019)	(0.008)	(0.031)
Unemployment rate (%) (t-1)	0.002	-0.001	0.005
	(0.003)	(0.001)	(0.004)
The number of employee (log) (t-1)	-0.006	-0.008*	0.013
	(0.010)	(0.005)	(0.016)
Weak instrument (Wald-F test)	333.361***		
Over-identification (Hansen’s J)	0.595	0.009	0.569
P-value	0.440	0.925	0.451
Adjusted R-squared	0.786	0.600	0.782

Notes: N = 132,445, Number of facilities = 13,754.

*** p<0.01

** p<0.05

* p<0.1.

Robust standard errors in parentheses (clustered by facility). All models control for facility–specific fixed effects, in addition to state and industry fixed effects and their interactions with linear year trends. For all models, the Hansen’s J statistics indicate the orthogonality of the instrumental variables (proportion of facilities adopting P2 in same industry and average number of inspections on other facilities in the state two years prior) cannot be rejected. Weak instrument test presents a statistically strong enough correlation between endogenous variable and IVs, given the Wald F statistic that is based on the Kleibergen–Paap rk statistics in the presence of clustered standard errors.

As another robustness check, we also include the estimated effects of *cumulative P2* on the levels of releases, amounts of wastes recycled, and amounts of wastes treated, separately (Table 6). Consistent with existing literature, we find that adopting more P2 techniques significantly reduces the average level of toxic releases and amounts of wastes treated. In addition, it does not affect the amounts of wastes recycled. Our findings suggest that estimating the effect of P2 on one pollutant at a time is unlikely to discover pollution substitution across categories. Specifically, even though adoption of P2 techniques reduced both releases and treated wastes on average, our results on the ratio of pollutants indicate that the reduction in releases is greater proportionally to its reduction in treatment and recycling, resulting net substitution between releases and other waste management methods.

**Table 6:** Second–Stage fixed–effects panel estimates on effects of P2 on levels of pollution.

Variable	Dependent Variables		
	(1)	(2)	(3)
	Log(treatment)	Log(recycling)	Log(total releases)
Cumulative P2 (t-1)	-0.274***	-0.078	-0.133**
	(0.094)	(0.099)	(0.058)
Total number of inspections (t-1)	0.0664***	0.039*	0.014
	(0.018)	(0.022)	(0.012)
PM nonattainment (t-1)	0.197**	0.030	-0.082
	(0.088)	(0.093)	(0.052)
Ozone nonattainment (t-1)	0.003	0.1744***	-0.073**
	(0.058)	(0.063)	(0.035)
Unemployment rate (%) (t-1)	0.0254***	0.0294***	-0.006
	(0.007)	(0.008)	(0.005)
Number of employees (log) (t-1)	0.1704***	0.1524***	0.0934***
	(0.034)	(0.038)	(0.019)
Production ratio (t-1)	0.1364***	0.1684***	0.2384***
	(0.035)	(0.037)	(0.023)
Weak instrument (Wald F-stat.)	30.7084***		
Over-identification (Hansen’s J)	1.126	1.936	0.0246
P-value of Hansen’s J	0.289	0.164	0.875
Observations	122,033	122,033	122,033
Number of Facilities	13,186	13,186	13,186
Adj. R-squared	0.773	0.771	0.783

Notes: Robust standard errors in parentheses (clustered by facility). All models control for facility specific fixed effects in addition to state and industry fixed effects and their interactions with linear year trends.

*** p<0.01

** p<0.05

* p<0.1.

For all models, the Hansen’s J statistics indicate the orthogonality of the instrumental variables (Proportion of facilities adopting P2 in same industry and average number of inspections on other facilities in the state two years prior) cannot be rejected. Weak instrument test presents a statistically strong enough correlation between endogenous variables and IVs, given the Wald F statistic based on the Kleibergen–Paap rk statistics in the presence of clustered standard errors.

Finally, we estimate Eq (2) using fixed-effects models without instrumental variables, thereby treating *cumulative P2* exogenous. The results are reported in Table 7. Without addressing the endogeneity of *cumulative P2*, its effect is attenuated (Table 7, row 1). As expected, the unobserved factors, such as adoption of environmental management systems, are likely to be correlated positively with the adoption of P2 techniques and negatively with pollution substitution, which leads to downward bias in fixed effects estimation without the use of IVs.

**Table 7:** Fixed–effects panel estimation on effects of P2 on pollution ratios without IVs.

Varibles	Dependent Variables		
	(1)	(2)	(3)
	Log(treatment/ Release)	Log(Water/Air)	Log(Recycling/ Releases)
Cumulative P2 (t-1)	0.001	0.000	-0.005*
	(0.002)	(0.001)	(0.003)
Total number of inspections (t-1)	0.011	-0.001	0.000
	(0.011)	(0.003)	(0.007)
PM nonattainment (t-1)	0.049	0.008	0.016
	(0.028)	(0.011)	(0.046)
Ozone nonattainment (t-1)	0.022	-0.003	0.092***
	(0.018)	(0.007)	(0.030)
Unemployment rate (%) (t-1)	-0.002	-0.002**	-0.002
	(0.003)	(0.001)	(0.004)
Number of employees (log) (t-1)	-0.001	-0.007	0.023
	(0.010)	(0.004)	(0.015)
Production ratio	0.047***	0.003	0.024
	(0.012)	(0.004)	(0.018)
Adj. R-squared	0.779	0.590	0.778

Notes: N = 130,133, Number of facilities = 13,925 Robust standard errors in parentheses (clustered by facility). All models control for facility–specific fixed effects, in addition to state and industry fixed effects and their interactions with linear year trends.

*** p<0.01

** p<0.05

* p<0.1.

## 5 Conclusions and discussion

The objective of this study is to examine the extent to which voluntary adoption of P2 techniques influences pollution substitution. Many studies have focused on the effect of regulatory pressure on pollution substitution or the effect of P2 adoption on reduction in toxic releases. This study contributes to the existing literature by providing the first empirical study on whether adopting more P2 techniques can reduce the overall use of toxics in the manufacturing industry.

Using data from 1991 to 2011, we set up three different types in pollution substitution as our dependent variables: the ratio of wastes treated to total releases, water releases to air releases, and wastes recycled to total releases. We use instrumental variables for the number of P2 techniques adopted and find that adopting greater numbers of P2 techniques does not influence the ratio between water releases and air releases. Previous studies find that adopting P2 techniques voluntarily leads to decreases in total toxic releases to the environment. Our results indicate this voluntary approach reduces all types of direct releases to the environment equally, thus curtailing pollution substitution often induced by regulations.

However, a voluntary policy to promote P2 is not a panacea to reduce total toxic wastes. We find that adopting greater numbers of P2 techniques contributes to increases in wastes emitted for treatment and recycling over total releases. Specifically, process and equipment modifications have a greater effect than do raw material, product, and procedure modifications. These results suggest that the potential of P2 techniques in reducing or eliminating overall reliance on toxics in manufacturing may be limited, as facilities focus on reducing releases to the environment through combining end-of-pipe and in-process waste management strategies with particular types of P2 techniques that do not necessarily address the root causes of toxic wastes. Thus, pollution control policy should emphasize waste minimization, considering the life cycle of toxics, and prioritize the use of raw material and product modification. As noted by Ranson et al. [6], raw material and product modifications are likely to be more resource intensive, thus grants and technical assistance programs should target them.

Additionally, our results have implications for other countries that are considering appropriate policies to promote pollution prevention. Following the example of TRI, the Organisation for Economic Co-operation and Development (OECD) has recommended that member countries establish reporting systems to track progress in pollution control and to facilitate exchanges of information on the best available techniques for P2 [43–45]. The establishment of the European Integrated Pollution Prevention and Control directive also recognizes the limitation of media-specific regulatory approaches and requires member countries to implement a more systematic environmental management to address the “cradle-to-grave” life cycle of toxic substances through the best available technologies while taking into account the heterogeneous environmental, technical, and economic conditions of the member countries. Our results indicate such an information system can be further improved by highlighting the adoption of waste minimization technologies by member countries.

There are several limitations associated with this study. First, our results apply to manufacturing facilities subject to the TRI reporting requirements over the period 1991 to 2011. Since TRI reporting facilities tend to be larger polluters, this may limit the generality of our findings on P2 programs implemented by other sectors, smaller businesses below the reporting thresholds, and more recently implemented P2 techniques. Second, recycling and treatment are still the preferred pollution control approaches over direct releases to the environment. More importantly, treatment and recycling methods reduce the toxicity of wastes released to the environment through stabilization, destruction, and reuse. Due to data limitation, it is difficult to incorporate chemical toxicity by calculating toxic-weighted emissions in this analysis since chemical toxicity differs by environment medium and by specific treatment and recycling method. Third, we focus on the average effect of P2 techniques without examining the effects of P2 techniques on pollution substitution by different industries due to data limitation. Future studies can conduct industry specific analysis through surveys to develop recommendations. For example, Gaona [46] shows that information on P2 techniques can be used to track green chemistry practices in the United States. Future analyses can extend our findings to chemical manufacturing to examine the effect of adopting P2 techniques related to green chemistry on pollution substitution.

## Supporting information

S1 File Lee_Bi_PONE2019. (ZIP)

## References

[1] Bi X. ‘Cleansing the air at the expense of waterways?’ Empirical evidence from the toxic releases of coal-fired power plants in the United States. J Regul Econ. 2017;51(1):18–40.

[2] Gibson M. Regulation-induced pollution substitution. Rev Econ Stat. 2019;forthcoming. Available from: 10.1162/rest_a_00797.

[3] Greenstone M. Estimating regulation-induced substitution: The effect of the Clean Air Act on water and ground pollution. Am Econ Rev. 2003;93(2):442–48. Available from: 10.1257/000282803321947498.

[4] King AA, Lenox MJ. Lean and green? An empirical examination of the relationship between lean production and environmental performance. Prod Oper Manage. 2001;10(3):244–56

[5] United States Environmental Protection Agency (EPA). Pollution prevention law and policies United States Environmental Protection Agency, Washington, DC; 2018a Available from: https://www.epa.gov/p2/pollution-prevention-law-and-policies#define.

[6] Ranson M, Cox B, Keenan C, Teitelbaum D. The impact of pollution prevention on toxic environmental releases from U.S. manufacturing facilities. Environ Sci Technol. 2015;49(21):12951–57. 10.1021/acs.est.5b02367 26477531

[7] Arora S, Gangopadhyay S. Toward a theoretical model of voluntary overcompliance. J Econ Behav Organ. 1995;28(3):289–309.

[8] Delmas M, Montes-Sancho MJ, Shimshack JP. Information disclosure polices: Evidence from the electricity industry. Econ Inquiry. 2010;48(2):483–98.

[9] Hamilton JT. Pollution as news: Media and stock market reactions to the toxics release inventory data. J Environ Econ Manage. 1995;28(1):98–113.

[10] Khanna M. Non-mandatory approaches to environmental protection. J Econ Surveys; 2001;15(3):291–324.

[11] Khanna M, Anton WRQ. Corporate environmental management: Regulatory and market-based incentives. Land Econ. 2002;78(4):539–58.

[12] Konar S, Cohen MA. Does the market value environmental performance? Rev Econ Stat. 2001;83(2):281–89.

[13] Harrington DR, Deltas G, Khanna M. Does pollution prevention reduce toxic releases? A dynamic panel data model. Land Econ. 2014;90(2):199–221.

[14] Alberini A. Environmental regulation and substitution between sources of pollution: An empirical analysis of Florida’s storage tanks. J Regul Econ. 2001;19(1):55–79.

[15] Berchicci L. Towards an Open R&D System: Internal R&D Investment, External Knowledge Acquisition and Innovative Performance. Res Policy. 2013; 42 (1): 117–127.

[16] Freeman H, Harten T, Springer J, Randall P, Curran MA, Stone K. Industrial pollution prevention! A critical review. J Air Waste Manage Assoc. 1992;42(5):618–56.

[17] Cagno E, Trucco P, Tardini L. Cleaner production and profitability: Analysis of 134 industrial pollution prevention (P2) project reports. J Cleaner Prod. 2005;13(6):593–605.

[18] Khanna M, Deltas G, Harrington DR. Adoption of pollution prevention techniques: The role of management systems, demand-side factors, and complementary assets. Environ Res Econ. 2007;44(1):85–106.

[19] Dahab MF, Montag DL, Parr JM. Pollution prevention and waste minimization at a galvanizing and electroplating facility. Water Sci Technol. 1994;30(5):243–50.

[20] Rajagopalan N, Boddu VM, Mishra S, Kraybill D. Pollution prevention in an aluminum grinding facility. Metal Finishing. 1998;96(11):18–24.

[21] Florida R, Davison D. Gaining from green management: Environmental management systems inside and outside the factory. California Manage Rev. 2001;43(3):64–84.

[22] Sam AG. Impact of government-sponsored pollution prevention practices on environmental compliance and enforcement: Evidence from a sample of U.S. manufacturing facilities. J Regul Econ. 2010;37(3):266–86.

[23] Bui LTM, Kapon S. The impact of voluntary programs on polluting behavior: Evidence from pollution prevention programs and toxic releases. J Environ Econ Manage. 2012;64(1):31–44.

[24] Bennear LS. Are management-based regulations effective? Evidence from state pollution prevention programs. J Policy Anal Manage. 2007;26(2):327–48.

[25] Sigman H. Cross-media pollution: Responses to restrictions on chlorinated solvent releases. Land Econ. 1996;72(3):298–312.

[26] Fullerton D, Karney DH. Multiple pollutants, co-benefits, and suboptimal environmental policies. J Environ Econ Manage. 2018;87:52–71.

[27] Holland SP. Taxes and trading versus intensity standards: Second-best environmental policies with incomplete regulation (leakage) or market power National Bureau of Economic Research, Cambridge, MA 2009;NBER 15262. Available from: https://www.nber.org/papers/w15262

[28] United States Environmental Protection Agency (EPA). Learn about the toxics release inventory United States Environmental Protection Agency, Washington, DC; 2018b Available from: https://www.epa.gov/toxics-release-inventory-tri-program/learn-about-toxics-release-inventory.

[29] Correia S. A feasible estimator for linear models with multi-way fixed effects; 2016 Available from: http://scorreia.com/research/hdfe.pdf.

[30] Burbidge JB, Magee L, Robb AL. Alternative transformations to handle extreme values of the dependent variable. J Am Stat Assoc. 1988;83(401):123–27.

[31] Hanna RN, Oliva P. The impact of inspections on plant-level air emissions. J Econ Anal Policy. 2010;10(1):Article-19.

[32] United States Environmental Protection Agency (EPA). NAAQS table United States Environmental Protection Agency, Washington, DC; 2018c Available from: https://www.epa.gov/criteria-air-pollutants/naaqs-table.

[33] United States Environmental Protection Agency (EPA). Nonattainment areas for criteria pollutants (green book) United States Environmental Protection Agency, Washington, DC; 2018d Available from: https://www.epa.gov/green-book.

[34] Walls & Associates. National establishment time-series (NETS) database: Database description; 2011.

[35] United States Bureau of Labor Statistics (BLS). Local area unemployment statistics United States Bureau of Labor Statistics, Washington, DC; 2011 Available from: https://www.bls.gov/lau/#tables.

[36] Harrington DR. Two-stage adoption of different types of pollution prevention (P2) activities. Res Energy Econ. 2012;34(3):349–73.

[37] Chang C, Sam AG. Corporate environmentalism and environmental innovation. J Environ Manage. 2015;153:84–92. 10.1016/j.jenvman.2015.01.010 25687809

[38] Baum CF, Stillman S, Schaffer ME. Enhanced routines for instrumental variables/GMM estimation and testing. Stata J. 2007;7(4):465–506.

[39] Dutt N, King AA. The judgment of garbage: End-of-pipe treatment and waste reduction. Manage Sci. 2014;60(7):1812–28.

[40] King A. Retrieving and transferring embodied data: Implications for the management of interdependence within organizations. Manage Sci. 1999;45(7):918–35.

[41] Rothenberg S. Knowledge content and worker participation in environmental management at NUMMI. J Manage Studies. 2003;40(7):1783–1802.

[42] Doshi AR, Dowell GW, Toffel MW. How firms respond to mandatory information disclosure. Strategic Manage J. 2013;34(10):1209–31.

[43] Koutalakis C, Buzogany A, Börzel TA. When soft regulation is not enough: The integrated pollution prevention and control directive of the European Union. Regul Gov. 2010;4(3):329–44.

[44] Organisation for Economic Co-operation and Development (OECD). Recommendation of the council on integrated pollution prevention and control Organisation for Economic Co-operation and Development, Paris, France; 2018a;OECD/LEGAL/0256.

[45] Organisation for Economic Co-operation and Development (OECD). Best available techniques for preventing and controlling industrial pollution Organisation for Economic Co-operation and Development, Paris, France; 2018b.

[46] Gaona SD. The utility of the toxic release inventory (TRI) in tracking implementation and environmental impact of industrial green chemistry practices in the United States. Green Chemistry; 2018 Available from: 10.5772/intechopen.70716

